# ZBP1 Drives IAV-Induced NLRP3 Inflammasome Activation and Lytic Cell Death, PANoptosis, Independent of the Necroptosis Executioner MLKL

**DOI:** 10.3390/v15112141

**Published:** 2023-10-24

**Authors:** R. K. Subbarao Malireddi, Bhesh Raj Sharma, Ratnakar R. Bynigeri, Yaqiu Wang, Jianlin Lu, Thirumala-Devi Kanneganti

**Affiliations:** Department of Immunology, St. Jude Children’s Research Hospital, 262 Danny Thomas Pl, Memphis, TN 38105, USA

**Keywords:** IAV, cell death, PANoptosis, necroptosis, inflammasome, MLKL, caspase, gasdermin, executioner, pyroptosis, apoptosis, NLRP3, RIPK3

## Abstract

Influenza A virus (IAV) continues to pose a significant global health threat, causing severe respiratory infections that result in substantial annual morbidity and mortality. Recent research highlights the pivotal role of innate immunity, cell death, and inflammation in exacerbating the severity of respiratory viral diseases. One key molecule in this process is ZBP1, a well-recognized innate immune sensor for IAV infection. Upon activation, ZBP1 triggers the formation of a PANoptosome complex containing ASC, caspase-8, and RIPK3, among other molecules, leading to inflammatory cell death, PANoptosis, and NLRP3 inflammasome activation for the maturation of IL-1β and IL-18. However, the role for other molecules in this process requires further evaluation. In this study, we investigated the role of MLKL in regulating IAV-induced cell death and NLRP3 inflammasome activation. Our data indicate IAV induced inflammatory cell death through the ZBP1-PANoptosome, where caspases and RIPKs serve as core components. However, IAV-induced lytic cell death was only partially dependent on RIPK3 at later timepoints and was fully independent of MLKL throughout all timepoints tested. Additionally, NLRP3 inflammasome activation was unaffected in MLKL-deficient cells, establishing that MLKL and MLKL-dependent necroptosis do not act upstream of NLRP3 inflammasome activation, IL-1β maturation, and lytic cell death during IAV infection.

## 1. Introduction

Influenza A virus (IAV) causes annual upper and lower respiratory infections world-wide, resulting in considerable morbidity, mortality, and economic burden [1]. The pathogenesis of IAV infection has been linked to severe lung inflammation, and immune-mediated lung inflammation and cell death are recognized as the primary factors responsible for IAV-induced pathology [2,3,4,5,6,7]. These cell death mechanisms, including IAV-induced cell death, have been extensively studied in the last decade and have been fundamental to the establishment of crosstalk, interconnections, and redundancies among cell death pathways that have been historically considered to be distinct [8]. Due to the molecular connections that have been found, IAV has emerged as an excellent model to study cell death.

Through the studies of IAV and cell death, the innate immune sensor Z-DNA binding protein 1 (ZBP1) has been identified as the key upstream sensor that is activated in response to IAV infection to induce cell death [9]. Mechanistically, upon activation, ZBP1 assembles a PANoptosome complex through heterotypic and homotypic domain interactions that promotes activation of both kinase and proteolytic signaling cascades to drive inflammation and lytic cell death [9,10,11,12]. The ZBP1-PANoptosome induces a form of cell death called PANoptosis, which is an inflammatory, lytic, innate immune programmed cell death pathway driven by RIPKs and caspases and regulated by PANoptosomes [13]. Loss of individual cell death effectors fails to provide significant protection from IAV-induced cell death, highlighting the functional redundancy among cell death components downstream of PANoptosome formation; in contrast, deletion of the key upstream sensor ZBP1 or simultaneous loss of the core PANoptosome components receptor interacting serine/threonine kinase 3 (RIPK3) and caspase-8 (CASP8) can protect bone marrow-derived macrophages (BMDMs) from IAV-induced PANoptosis [9].

While it is well-established that ZBP1 acts as an innate immune sensor of IAV infection to induce robust inflammatory cell death, PANoptosis, in infected macrophages, the downstream events involved in executing ZBP1-dependent PANoptosis require further investigation [9,10,11,14,15,16]. Early studies have shown that IAV induces NLR family pyrin domain containing 3 (NLRP3)-dependent inflammasome activation and IL-1β and IL-18 maturation, but genetic deletion of NLRP3 fails to protect the cells from death [9]. Similarly, these studies have shown that IAV also induces mixed lineage kinase domain-like (MLKL) protein activation, which is generally indicative of necroptosis, but loss of MLKL fails to provide protection from IAV-induced cell death [9,12], suggesting this is not necroptosis. However, other reports showed that MLKL activation may instead act as a standalone mechanism, and loss of MLKL provides significant delay and protection from IAV-induced cell death and inflammasome activation [14,15,16,17]. Therefore, the role of MLKL in IAV-induced cell death remains unclear. To address these discrepancies, in this study, we investigated the role of MLKL in regulating IAV-induced cell death and NLRP3 inflammasome activation. We found that both IAV-induced cell death and NLRP3 inflammasome activation were fully independent of MLKL in macrophages. Furthermore, we observed IAV induced ZBP1-dependent, but RIPK3-independent PANoptosis at extended timepoints post-IAV infection in macrophages, suggesting that RIPK3 became redundant with other cell death molecules at later timepoints. Overall, our data clarify the role of MLKL in IAV-induced cell death and provide key insights for strategies to target cell death to limit IAV immunopathogenesis.

## 2. Materials and Methods

### 2.1. Mice

*Zbp1*^−/−^ [18], *Ripk3*^−/−^ [19], *Ripk3*^−/−^*Casp8*^−/−^ [20], *Mlkl*^−/−^ [21], and *Nlrp3*^−/−^ [22] mice have been reported previously, and C57/BL6J control mice (WT) used in the current study were bred at the Animal Resources Center at St. Jude Children’s Research Hospital and maintained under specific pathogen-free conditions. Mice were maintained on a 12-h light/dark cycle and were fed standard chow. Animal studies were conducted under protocols approved by the St. Jude Children’s Research Hospital Committee on the Use and Care of Animals (IACUC protocol, #482).

### 2.2. Primary Macrophage Differentiation and Stimulation

Freshly isolated murine bone marrow cells were plated into three 150 mm tissue culture plates, each containing a total of 20 mL of macrophage differentiation medium (BMDM medium). BMDM medium was prepared by supplementing IMDM (12440053, Thermo Fisher Scientific, Waltham, USA) with 30% L929 cell-conditioned medium, 10% heat inactivated (HI)-FBS, 1% nonessential amino acids (11140-050, Thermo Fisher Scientific), and 1% penicillin–streptomycin (15070-063, Thermo Fisher Scientific). An additional 5 mL of fresh BMDM medium was added to each plate on d 3 and 5 to provide better growth and differentiation conditions. The fully matured macrophages were collected and counted on d 6, followed by seeding at 10^6^ cells per well in 12-well culture plates in DMEM containing 10% HI-FBS and 1% penicillin–streptomycin for the experimental stimulations on the following day. Infections were performed with IAV (20 moi) for the indicated timepoints, and the infected BMDMs were treated with the pharmacological MLKL inhibitor GW806742X (MedChemExpress, Monmouth Junction, NJ, USA, 1 μM final concentration), or the RIPK1 inhibitor Nec1s (Selleckchem, Houston, TX, USA, 25 μM final concentration), starting from 1 h post-infection, as detailed in the figure legends.

### 2.3. Virus Stock Preparation

The IAV (A/Puerto Rico/8/34, H1N1 [PR8]) was prepared as previously described [12] and propagated from 11-d-old embryonated chicken eggs by allantoic inoculation. The IAV titer was measured by plaque assay in Madin–Darby canine kidney (MDCK) cells.

### 2.4. Western Blotting

The samples for Western blot analyses were prepared as described previously [23]. For caspase immunoblotting, the samples were prepared by combining the cell lysates with culture supernatants (lysis buffer: 5% NP-40 solution in water supplemented with 10 mM DTT and protease inhibitor solution at 1× final concentration). The lysate samples for immunoblot analysis of all other proteins were prepared by lysing the cells in RIPA buffer, without combining with the supernatants. All samples were mixed and denatured in loading buffer containing SDS and 100 mM DTT and boiled for 12 min. SDS-PAGE-separated proteins were transferred to PVDF membranes (IPVH00010, Millipore, Burlington, NJ, USA) using the Trans-Blot^®^ Turbo™ system. Immunoblotting was performed with primary antibodies against caspase-1 (AG-20B-0042; Adipogen, San Diego, CA, USA, 1:1000), caspase-3 (#9662, Cell Signaling Technology [CST], Danvers, MA, USA, 1:1000), cleaved caspase-3 (#9661, CST, 1:1000), caspase-7 (#9492, CST, 1:1000), cleaved caspase-7 (#9491, CST, 1:1000), caspase-8 (#4927, CST, 1:1000), cleaved caspase-8 (#8592, CST, 1:1000), GSDMD (ab209845, Abcam, Waltham, MA, USA, 1:1000), GSDME (ab19859, Abcam, 1:1000), pMLKL (#37333, CST, 1:1000), tMLKL (AP14242B, Abgent, San Diego, CA, USA, 1:1000), and β-Actin (sc-47778 HRP, Santa Cruz, Dallas, TX, USA, 1:5000). Appropriate horseradish peroxidase (HRP)-conjugated secondary antibodies (anti-mouse [315-035-047] and anti-rabbit [111-035-047], Jackson ImmunoResearch Laboratories, West Grove, PA, USA) were used as described previously [24]. Immunoblot images were acquired on an Amersham Imager using Immobilon^®^ Forte Western HRP Substrate (WBLUF0500, Millipore).

### 2.5. Cell Death Analysis

As described previously [24], time course analyses of cell death were performed using a two-color IncuCyte S3/SX5 incubator imaging system. Fully differentiated BMDMs were seeded in 12-well (1.0 × 10^6^ cells/well) or 24-well (0.5 × 10^6^ cells/well) tissue culture plates in the presence of propidium iodide (PI) (P3566, Life Technologies, Carlsbad, CA, USA), which leaks into the dying cells and marks them as positive for lytic cell death. The cells were infected with IAV at 20 moi, as indicated in the figure legends. Time course image acquisition was carried out using the 20× objective, and the dead cells positive for PI-uptake were marked by the mask-application provided in the IncuCyte software modules S3 and SX5. A minimum of four images per well for each condition and timepoint were acquired for the quantitative studies. The dead cells positive for PI-uptake were pseudo-colored in red for the presentation of the original imaging data in the figures. The percent dead cell counts were plotted using GraphPad Prism version 9.0 software.

### 2.6. ELISA Analyses

The quantification of the secreted cytokines from the cell culture supernatants was performed as per the protocols provided by the commercial Milliplex kit supplier, Millipore (#MCYTOMAG-70K).

### 2.7. Statistical Analyses

GraphPad Prism version 8.0 and 9.0 software packages were used for data analyses. Data are presented as mean ± SEM. Statistical significance was determined by Student’s *t*-test for two groups or a one-way ANOVA for two or more groups (Dunnett’s multiple comparisons test).

## 3. Results

### 3.1. Loss of Necroptosis Executioner MLKL Does Not Protect Macrophages from IAV-Induced Cell Death

ZBP1, also known as DAI (DNA-dependent activator of IFN regulatory factors), has emerged as the critical innate immune sensor of IAV infection [9]. Previous studies have demonstrated that activated ZBP1 serves as a master regulator of IAV-induced NLRP3 inflammasome activation and inflammatory cell death, PANoptosis [9,10,12]. PANoptosis is a unique innate immune, lytic and inflammatory cell death pathway driven by caspases and RIPKs that is regulated by the multiprotein PANoptosome complex [25]. The IAV-induced ZBP1-PANoptosome contains several components, including RIPK3 and CASP8 [9,10,12]. Previous studies demonstrated that genetic deletion or inhibition of individual components of IAV-induced PANoptosis is not sufficient to rescue the cells from undergoing cell death [9,12]. However, contrasting reports have suggested the possibility that IAV-induced necroptosis may act as a standalone, non-redundant mechanism of cell death and inflammasome activation downstream of ZBP1-RIPK3 signaling [14,15,16,17], leaving the current state of IAV-induced cell death mechanisms unclear. To address this gap in our understanding, we sought to use macrophages as a model system to address the role of MLKL in IAV-induced cell death and inflammasome activation. Fully differentiated BMDMs from WT, *Zbp1*^−/−^ (ZBP1 knockout (KO)), *Ripk3*^−/−^ (RIPK3 KO), and *Mlkl*^−/−^ (MLKL KO) mice were infected with IAV. Then the dynamics and magnitude of the cell death was monitored using IncuCyte-based live imaging analyses, where the dead cells were marked by PI (propidium iodide) dye uptake upon the loss of membrane integrity.

**Figure 1:**
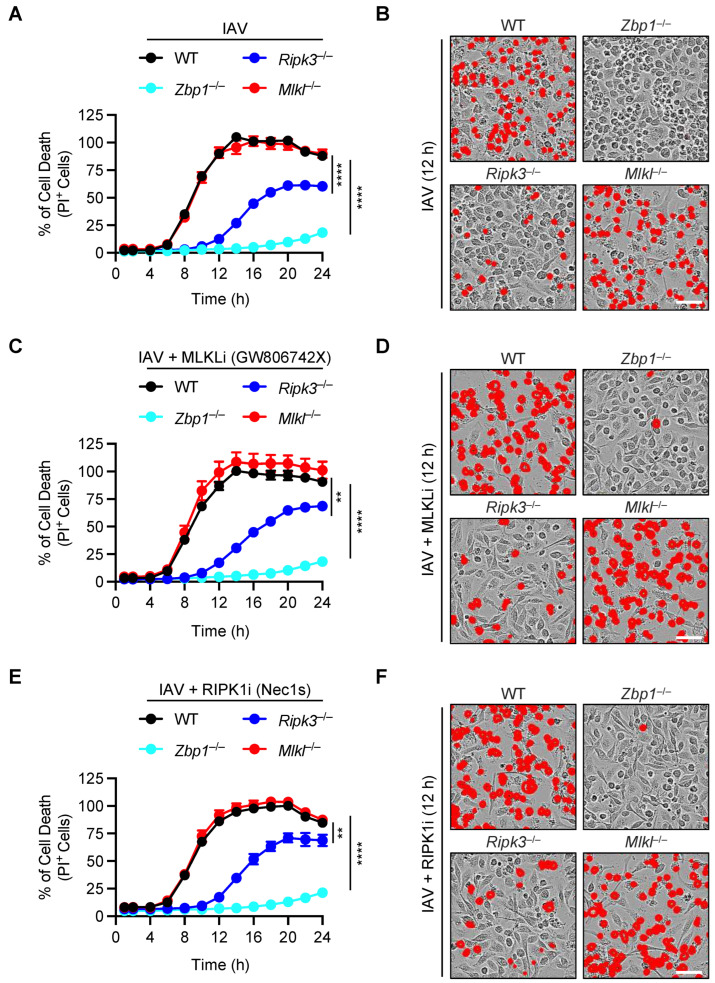
**Loss of MLKL does not protect macrophages from IAV-induced cell death.** (**A**–**F**) Analysis of cell death in IAV-infected wild-type (WT) and different gene-deficient bone marrow-derived macrophages (BMDM) as measured by IncuCyte-based image analyses. Quantification and time course analysis of cell death in WT, *Zbp1*^−/−^, *Mlkl*^−/−^ and *Ripk3*^−/−^ BMDMs infected with IAV (**A**), infected with IAV plus treated with the MLKL inhibitor (MLKLi, GW806742X, 1 μM final concentration) (**C**), and infected with IAV plus treated with the RIPK1 inhibitor (Nec1s, 25 μM final concentration) (**E**). Representative images of cell death collected at 12 h post-infection from WT, *Zbp1*^−/−^, *Mlkl*^−/−^, and *Ripk3*^−/−^ BMDMs infected with IAV (**B**), infected with IAV plus treated with the MLKL inhibitor (MLKLi, GW806742X, 1 μM final concentration) (**D**), and infected with IAV plus treated with the RIPK1 inhibitor (Nec1s, 25 μM final concentration) (**F**). The RIPK1 and MLKL inhibitors were added 1 h post-infection (**C**–**F**). The data are representative of at least three independent experiments (**A**–**F**). The red mask denotes the dead cells, and the scale bar represents 50 μM (**B**,**D**,**F**). ** *p* < 0.01; **** *p* < 0.0001. A one-way ANOVA (Dunnett’s multiple comparisons test) was used (**A**,**C**,**E**). The data are represented as mean ± SEM (**A**,**C**,**E**).

We observed that IAV-induced robust cell death in wild-type (WT) macrophages, and the genetic deletion of ZBP1 rescued the cells from IAV-induced cell death (Figure 1A,B). In contrast, we found that loss of RIPK3 provided partial protection from cell death and failed to phenocopy the ZBP1-deficient cells (Figure 1A,B); this contradicts the existing concept that the ZBP1-RIPK3 axis is the key for driving IAV-induced cell death [9,12,14,15,16]. Moreover, there were key differences between RIPK3-deficient cells and MLKL-deficient cells. The *Mlkl*^−/−^ BMDMs showed similar cell death kinetics and magnitude to that of WT cells and failed to phenocopy the delay in cell death observed in *Ripk3*^−/−^ BMDMs (Figure 1A,B). Together, these results suggest that IAV-induced cell death is dependent on ZBP1 but not MLKL or its upstream activating kinase RIPK3.

To provide an additional line of evidence to understand the roles of MLKL and RIPK3 in IAV-induced cell death, we next treated WT, ZBP1 KO, RIPK3 KO, and MLKL KO cells with the MLKL inhibitor (MLKLi, GW806742X), starting from 1 h post-IAV infection. Consistent with our results from gene-deficient BMDMs (Figure 1A,B), we found that MLKL inhibitor treatment did not affect the IAV-induced cell death in either WT BMDMs or other gene-deficient cells, indicating that MLKL inhibition did not block the IAV-induced cell death (Figure 1C,D). Furthermore, we used a complementary approach of inhibiting necroptosis using the well-established RIPK1 kinase inhibitor, necrostatin-1s (Nec1s). Treatment with Nec1s also did not block the IAV-induced cell death (Figure 1E,F). Together, these data demonstrated that ZBP1 is the master regulator of IAV-induced cell death in BMDMs and that RIPK3 partially contributes to this process. Moreover, our data demonstrate that MLKL is not required for IAV-induced cell death.

### 3.2. Loss of MLKL Does Not Protect Macrophages from IAV-Induced Biochemical Activation of PANoptosis

**Figure 2:**
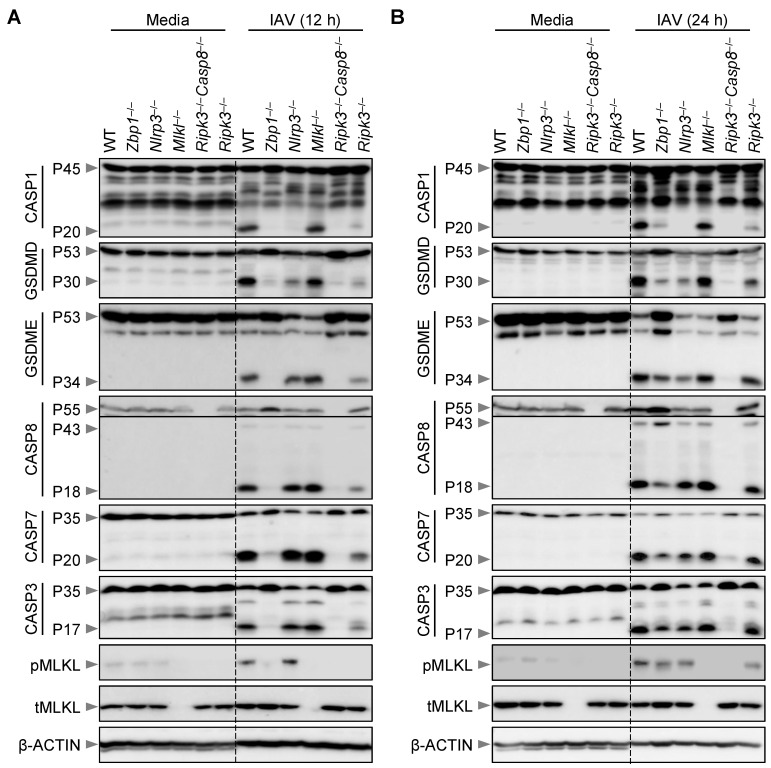
**Loss of MLKL does not protect macrophages from IAV-induced activation of PANoptosis molecules.** (**A**,**B**) Western blot analysis of PANoptosis in IAV-infected wild-type (WT) and different gene-deficient bone marrow-derived macrophages (BMDMs). Western blot analysis of pro- (P45) and activated (P20) caspase-1 (CASP1), pro- (P53) and activated (P30) gasdermin D (GSDMD), pro- (P53) and activated (P34) gasdermin E (GSDME), pro- (P55) and cleaved (P43 and P18) caspase-8 (CASP8), pro- (P35) and cleaved (P20) caspase-7 (CASP7), pro- (P35) and cleaved (P17) caspase-3 (CASP3), phosphorylated MLKL (pMLKL) and total MLKL (tMLKL) in WT, *Zbp1*^−/−^, *Nlrp3*^−/−^, *Mlkl*^−/−^, *Ripk3*^−/−^*Casp8*^−/−^, and *Ripk3*^−/−^ BMDMs at 12 h (**A**) and 24 h (**B**) post-infection with IAV. The data are representative of at least three independent experiments (**A**,**B**). The uppercase ’P’ in the Western blots represents the molecular weight of the proteins. The lowercase letters ‘p’ and ‘t’ represent the phospho- and total-forms of the proteins, respectively.

Real-time IncuCyte imaging analysis demonstrated that IAV induced robust ZBP1-dependent lytic cell death in BMDMs (Figure 1). Previous studies have established that the IAV-induced cell death modality can be biochemically characterized by the activation of the NLRP3 inflammasome as well as caspases and RIPKs, which are biochemical features of PANoptosis [9,12]. However, contrasting reports have suggested that the IAV-induced activation of the NLRP3 inflammasome and cell death molecules occur, at least in part, in a necroptosis-dependent manner [14,15,16,17]. Therefore, we next sought to address whether MLKL can also contribute to the inflammasome and PANoptosis molecule activation at the biochemical level in unprimed IAV-infected BMDMs. Using immunoblotting-based biochemical analyses, we observed IAV-induced PANoptosis in BMDMs at both 12 and 24 h post-infection, as indicated by activation of CASP1, gasdermin D (GSDMD) and GSDME, along with CASP8, CASP3, and CASP7, as well as MLKL (Figure 2A,B). Consistent with our cell death data (Figure 1), we found that the activation of these PANoptosis molecules was completely abrogated at 12 h post-infection in IAV-infected *Zbp1*^−/−^ BMDMs, and we observed a similar phenotype in *Ripk3*^−/−^*Casp8*^−/−^ BMDMs (Figure 2A). At the same early time of 12 h post-infection, the RIPK3 KO BMDMs started to diverge from the ZBP1 KO cells and began to show the activation of caspases and GSDMD and GSDME (Figure 2A). Furthermore, the immunoblotting data showed normal activation of CASP1 in *Mlkl*^−/−^ cells, but CASP1 activation was fully abrogated in NLRP3 KO cells at this early timepoint (Figure 2A).

At 24 h post-IAV infection, we observed robust PANoptosis in WT BMDMs, which was substantially reduced in ZBP1-deficient cells, although not fully abrogated; this was in contrast to *Ripk3*^−/−^*Casp8*^−/−^ BMDMs, where activation of the PANoptosis molecules was still fully inhibited at 24 h post-IAV infection (Figure 2B). At the same late time of 24 h post-infection, the *Ripk3*^−/−^ BMDMs showed almost WT levels of activation of many PANoptosis molecules, consistent with the cell death phenotype we observed; however, *Ripk3*^−/−^ BMDMs continued to show significantly reduced CASP1 and GSDMD activation at the 24 h timepoint (Figure 2B). We also observed MLKL activation that was independent of the ZBP1-RIPK3-axis that began to emerge at this late timepoint of 24 h post-IAV infection (Figure 2B), which suggests that the loss of cellular homeostasis and/or accumulation of cellular stress could lead to activation of other compensatory cell death pathways.

Additionally, loss of MLKL failed to block the activation of PANoptosis molecules, including activation of the inflammasome components CASP1 and GSDMD, both at 12 h and 24 h post-IAV infection (Figure 2A,B). Collectively, immunoblotting analyses demonstrated that ZBP1 is the master regulator of RIPK3-CASP8-dependent NLRP3 inflammasome activation and PANoptosis, whereas the downstream MLKL does not act as a standalone mechanism during IAV infection under non-priming conditions.

### 3.3. MLKL Is Not Required for IAV-Induced Inflammatory or Inflammasome-Dependent Cytokine Production

**Figure 3:**
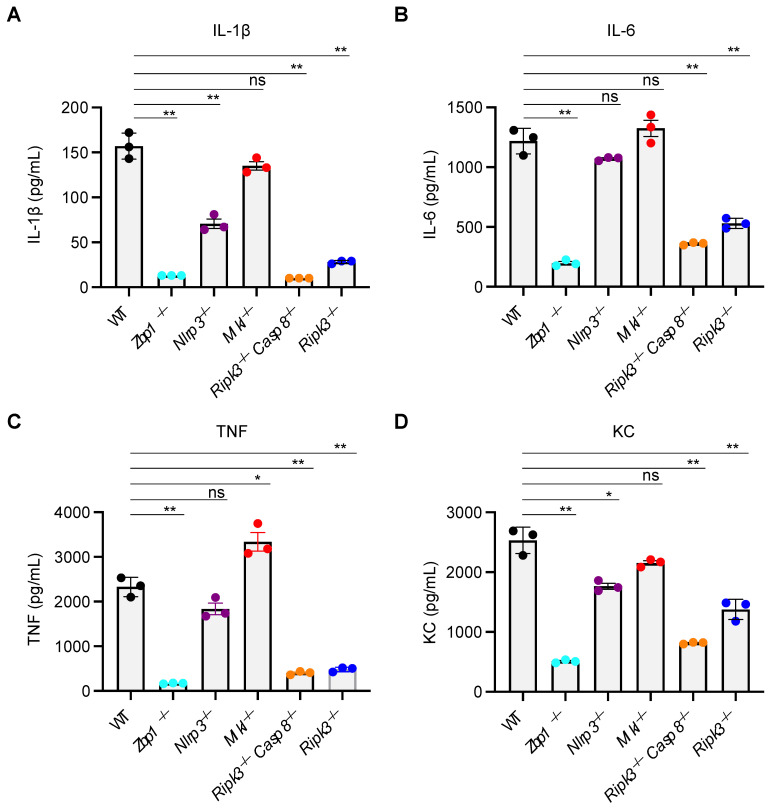
**MLKL is not required for IAV-induced inflammatory or inflammasome-dependent cytokine production.** (**A**–**D**) ELISA-based analysis of cytokines in IAV-infected wild-type (WT) and different gene-deficient bone marrow-derived macrophages (BMDMs). Quantification of cytokines IL-1β (**A**), IL-6 (**B**), TNF (**C**), and KC (**D**) in supernatants collected from IAV-infected WT, *Zbp1*^−/−^, *Nlrp3*^−/−^, *Mlkl*^−/−^, *Ripk3*^−/−^*Casp8*^−/−^, and *Ripk3*^−/−^ BMDMs at 24 h post-infection. The data are representative of at least three independent experiments (**A**–**D**). ns, not significant, * *p* < 0.05; ** *p* < 0.01. The Student’s *t*-test was used (**A**–**D**). The data are represented as mean ± SEM (**A**–**D**).

Since expression, activation, and release of soluble inflammatory mediators are key features of inflammatory cell death and PANoptosis [9,12], we next sought to address the role of MLKL and other PANoptosis regulators in cytokine secretion at 24 h post-IAV infection. Loss of ZBP1, NLRP3, RIPK3, and RIPK3 plus CASP8 proteins caused significant reductions in the release of the IAV-induced inflammasome-dependent cytokine interleukin (IL)-1β (IL-1β), but loss of MLKL failed to affect the levels of IL-1β produced (Figure 3A). Analyses of inflammasome-independent cytokines, including IL-6, TNF, and the chemokine KC (IL-8), also demonstrated that ZBP1, RIPK3, and CASP8 are required for the release of non-inflammasome cytokines, whereas NLRP3 and MLKL are generally not required for these processes during IAV infection (Figure 3B–D). Together, these results indicate that the ZBP1-RIPK3 axis, independent of MLKL, regulates inflammatory cell death, PANoptosis, and the expression of inflammasome-independent inflammatory cytokines and chemokines, as well as inflammasome-dependent, CASP1-mediated cytokine secretion during IAV infection in BMDMs.

## 4. Discussion

In this study, we confirmed the previous findings that ZBP1 senses IAV in BMDMs and drives PANoptosis [9], and we found that this is partially dependent on RIPK3, but fully abrogated in *Ripk3*^−/−^*Casp8*^−/−^ cells. Pharmacological inhibition or genetic deletion of MLKL did not result in either reduced cell death or decreased biochemical activation of the inflammasome or PANoptosis. These findings demonstrate that MLKL does not act as a standalone mechanism of cell death at phenotypic or biochemical levels during IAV infection in unprimed primary BMDMs. These results are important in the context of inflammasome-dependent inflammatory cytokine activation, which plays crucial roles in further amplification of innate immune responses and shaping adaptive immune responses [26]. Studies showing a critical protective role for the inflammasome [27], but not for MLKL [16,17,28], in IAV-infected animals further support the concept that MLKL is dispensable for the inflammasome’s protective functions during IAV infection in vivo. Our data support a model where loss of the RIPK3-MLKL axis is not sufficient to block IAV-induced inflammasome activation and IL-1β secretion, and that ZBP1-mediated PANoptosis acts as a versatile host defense mechanism against IAV infection [9,10,12]. However, a recent study showed an independent role for MLKL in driving IAV-induced cell death and inflammasome activation, particularly early in the infection cycle, in the context of TLR priming [17]. Our data suggest that the phenotype observed in that study is dependent on TLR priming rather than on the viral infection. Additionally, a previous study suggested that GSDMD activation could be an upstream regulator of necroptosis by showing that mitochondrial-ROS can promote GSDMD activation in macrophages with the disease-associated gain-of-function allele Lrrk2^G2019S^ (leucine-rich repeat kinase 2), which in turn acts as an upstream trigger driving MLKL-dependent necroptosis [29]. However, in our study, no such cross-regulation was observed in IAV-infected BMDMs, as we found normal activation of phosphorylated MLKL in *Nlrp3*^−/−^ cells, despite the lack of GSDMD activation in those cells. Together, our findings suggest that necroptosis may be limited as a fail-safe mechanism of inflammatory cell death that is often activated upon CASP8 inactivation and may be a poor activator of inflammasome-dependent cytokines during IAV infection.

As with any other pathogen, innate immunity acts as the first line of defense against influenza infections. The viral infection of epithelial cells and macrophages triggers inflammatory cell death responses, which appear to be vital for viral clearance, but their uncontrolled or excessive activation can promote pathological tissue damage, leading to the development of acute respiratory distress syndrome (ARDS)-like disease, which is responsible for respiratory and multiorgan failure and can even cause death of the infected patients [6,30]. It is increasingly clear that exaggerated activation of innate immune components is responsible for the development of severe lung damage. Therefore, these molecules are viable therapeutic targets to mitigate the pathology in combination with antiviral drugs [31,32,33]. However, caution is warranted, as it is essential to delineate the protective versus detrimental innate immune responses and develop specific targeting strategies to avoid uncontrolled viral replication [31,32,33]. Moreover, it is now clear that different strains of IAV differ in replication efficiencies and ability to evade the host anti-viral immune responses. For example, studies using the mouse adapted PR8 H1N1 strain have shown that it has high pathogenicity in vivo but reduced infectivity in macrophages compared to other IAV strains such as H3N2 and the seasonal H1N1 variants [34,35,36]. These observations are important in the context of avian and other zoonotic infections, as these strains represent emerging pathogenic IAV strains that are capable of infecting and replicating in human cells, with the potential to cause pandemics via human-to-human community transmissions and excessive activation of inflammatory immunopathology [37,38,39,40,41,42,43]. Together, these studies further support the concept that uncontrolled inflammation and viral replication are responsible for increased mortality and morbidity in cases of severe influenza, and other viral, infections. In line with these observations, the loss of receptors for the inflammatory cytokines TNF and IL-1 reduces lung inflammation and extends the survival of influenza-infected mice [44]. Furthermore, recent studies have shown that proinflammatory cytokines, especially TNF when combined with IFNγ, can trigger robust PANoptosis in various cell types, which also drives tissue damage and mortality in SARS-CoV-2 infections [45,46,47,48,49]. In this regard, it is important for future studies to determine whether the highly pathogenic influenza strains are more like SARS-CoV-2 in their ability to evade the primary innate immune responses and induce activation of robust secondary inflammatory cytokine responses, leading to cytokine storms and the induction of detrimental PANoptosis.

Beyond the specific implications for influenza infections, ZBP1 has been found to be involved in other infections [46,50], and several genetic studies have demonstrated that activated PANoptosome components are often associated with hyperactivation of ZBP1 or inflammasome assembly and IL-1β secretion in diverse pathophysiological conditions [51,52,53,54,55,56,57,58,59,60,61]. The sum of data from our group and others provides a compelling rationale for therapeutic targeting of ZBP1 as an improved strategy for modulating immune responses in the context of infectious and inflammatory disease conditions. Future research aimed at deciphering the intricate cross-regulatory mechanisms that activate complementary cell death modalities and identifying key upstream sensor molecules similar to ZBP1 may aid in the development of therapeutic agents with higher efficacy and minimal toxicity.

## Data Availability

The data generated and presented in the current study are provided within the manuscript. Correspondence and requests for materials should be addressed to thirumala-devi.kanneganti@stjude.org.
